# Mechanisms of the formation of psychological disadaptation in combatants with a distinction based on the signs of adjustment disorders and PTSD

**DOI:** 10.1192/j.eurpsy.2023.2076

**Published:** 2023-07-19

**Authors:** B. Mykhaylov, V. Mishiev, E. Grinevich, V. Omeljanovich, O. Kudinova

**Affiliations:** 1 National Health Care University named Shupik, Kyiv; 2 Kharkiv Medical Academy Postgraduate Education, Kharkiv, Ukraine

## Abstract

**Introduction:**

Contemporary for combatants with signs of adjustment disorders (AD) consists of “distorted” thoughts about the displacement of the value of one’s role, the loss of a certain significant position. This can be seen from the analysis of complaints about the feeling of anxiety, tension, asthenia, mood swings, lack of emotional regulation and the assessment of well-being criteria.

It is important to understand what is a preventer of maladjustment, and what is a predictor of the formation of psychological adaptation disturbances in combatants with a distinction based on the signs of AD and PTSD.

**Objectives:**

Based on the analysis of violations of the psycho-emotional sphere, peculiarities of perception of the social environment, understanding of one’s role in traumatic events, and personal adaptation potential, predictors and preventers of violation of psychological adaptation among combatants are determined.

**Methods:**

The clinical investigation based on psychiatric examination with the narrative motivation interview,psychological examination by Mississippi Scale for Combat-Related Posttraumatic Stress Disorder.

**Results:**

The predictors of impaired psychological adaptation in combatants withAD: Low indicators of moral normativity, which structure thoughts and help in the interpretation and analysis of negative experienced events of a stressogenic level.Preventers of violation of psychological adaptation in combatants with AD:Communicative potential, which lies in the structure of personal adaptation potential. This indicator indicates the presence of a need in the social environment and opportunities for realization, that is, as a resource of the individual. The ability to receive from the social environment a sense of support, reinforcement of self-esteem, motivation for activity, determination of the goals of future projects. Predictors of impaired psychological adaptation in combatants with PTSD: Low communicative potential, which, under the significant influence of stressogenic factors, isolates from close social interaction and does not allow family members to influence the emotional state. Family support is diminished and perceived as a trigger for anger. Fixation on the intensity of emotional experiences, maintaining the tone of negative manifestations, as a form of receiving punishment. Keeping under control one’s values in the life system and an attempt to reorient oneself to the future.

**Conclusions:**

Relying on preventers as the resource base of the personality of combatants and predictors as targets of psychocorrective intervention in the tasks of the medical and psychological rehabilitation program, algorithms for further psychocorrective intervention were determined.

**Disclosure of Interest:**

None Declared

